# *Mycobacterium tuberculosis* Complex Drug Resistance in Italy

**DOI:** 10.3201/eid1004.030667

**Published:** 2004-04

**Authors:** Giovanni B. Migliori, Rosella Centis, Lanfranco Fattorini, Giorgio Besozzi, Cesare Saltini, Claudio Scarparo, Daniela Cirillo, A. Gori, Antonio Cassone, Claudio Piersimoni

**Affiliations:** * SMIRA (Italian Multicentre Study on Resistance to Antituberculosis drugs) Coordinating Committee

**Keywords:** tuberculosis, prevalence of drug resistance, drug susceptibility testing

**To the Editor:** The reemergence of tuberculosis (TB) as a global health problem over the past 2 decades, accompanied by increased drug resistance, which represents a serious problem both in terms of TB control and clinical management (*1*), prompted Western European countries to develop comprehensive national surveillance systems to monitor trends in TB drug resistance. Moreover, the World Health Organization (WHO) and the International Union Against Tuberculosis and Lung Disease (IUATLD) launched the Global Project on Anti-Tuberculosis Drug Resistance Surveillance to measure the prevalence of drug resistance by using standardized methods and assess its correlation with indicators of TB control (*2*,*3*). Since comprehensive data on resistance to firstline drugs were not available in Italy, a network of 20 regional laboratories was established to participate in this project. The Department of Bacteriology and Medical Mycology of Istituto Superiore di Sanità in Rome and the Mycobacteriology Unit of Istituto Villa Marelli in Milan (appointed respectively as Supranational Reference Laboratory and National Reference Laboratory) supervised and controlled the network of regional laboratories. The combination of reference laboratories in the network and associated clinical units, which covered 30% of definite cases reported each year (*4*), was known as SMIRA (Italian Multicentre Study on Resistance to Anti-Tuberculosis Drugs). The WHO/IUATLD coordinating center in Ottawa, Canada, provided a batch of 20 *Mycobacterium tuberculosis* strains to set up proficiency testing to check drug susceptibility procedures in all SMIRA laboratories (*5*). We summarize the nature and extent of TB drug resistance in Italy between 1998 and 2001.

Isolates from all consecutive, definite cases diagnosed in TB units during 1998 through 2001 were included. When a patient’s previous treatment status was unknown or dubious, the case was excluded. Resistant cases from patients with and without history of previous treatment were stratified by the following categories: any resistance, monoresistance, resistance to both isoniazid and rifampicin (known as rifampin in the United States), or resistance to three or more drugs. Confidence intervals were also calculated. Participating laboratories were allowed to use the WHO-recommended drug susceptibility method with which they were most familiar: absolute concentration method, resistance ratio method, proportion method and its variants, or BACTEC 460 radiometric method (Becton Dickinson, Towson, MD) (*6*,*7*). Among the laboratories reporting results by the proportion method, the majority used Löwenstein-Jensen medium while others used liquid nonradiometric media (*8*). Each of the 20 *M. tuberculosis* strains was tested against firstline drugs by the Italian Reference Laboratories in Rome and Milan and classified as resistant or susceptible. Results were compared to the standard criterion, represented by the judicial results of the WHO/IUATLD Global Network of Supranational Laboratories (*9*). Each network laboratory was validated for each firstline drug when no more than two results were different from the standard criterion.

The prevalence of drug resistance detected during the period 1998–2001 is summarized in the Table. Among previously untreated cases, the prevalence of resistance to isoniazid, rifampicin, ethambutol, and streptomycin was 3.5%, 0.8%, 0.5%, and 4.3%, respectively, while prevalence of multidrug resistance (resistance to at least isoniazid and rifampicin) and polyresistance (resistance to two or more drugs, but not both isoniazid and rifampicin) was 1.1% and 2.4%, respectively. No difference was found by stratifying prevalence data by age, sex, or HIV status. In isolates from patients with previous treatment, drug resistance was found to be almost four times higher than in those from patients with no history of treatment. However, the prevalence of monoresistant strains was low (5.3%, 4.3%, 0.3%, and 4.3% for isoniazid, rifampicin, ethambutol, and streptomycin, respectively) compared with the prevalence of multidrug-resistant strains whose rate reached a peak of 30.4%.

**Table:** Pattern of drug resistance among strains from tuberculosis patients with and without a history of treatment, Italy 1998–2001^a^

Tested MTB strains	No history of previous treatment			History of previous treatment		
	No.	%	95% CI	No.	%	95% CI
Total tested	2,117	100	**–**	322	100	**–**
Fully sensitive	1,847	87.2	85.8 to 88.6	155	48.1	42.7 to 53.6
Any drug	270	12.7	11.4 to 14.2	167	51.8	46.4 to 57.3
INH	75	3.5	2.8 to 4.4	17	5.3	3.2 to 8.2
RMP	17	0.8	0.5 to 1.3	14	4.3	2.5 to 7.0
EMB	10	0.5	0.2 to 0.8	1	0.3	0.02 to 1.5
SM	93	4.3	3.6 to 5.3	14	4.3	2.5 to 7.0
Resistant to both INH and RMP	8	0.40	0.8 to 0.7	24	7.5	4.9 to 10.7
Resistant to INH, RMP, EMB	2	0.10	0.01 to 0.3	19	6.0	3.7 to 8.9
Resistant to INH, RMP, SM	6	0.30	0.1 to 0.6	23	7.1	4.7 to 10.4
Resistant to INH, RMP, EMB, SM	7	0.30	0.1 to 0.6	32	9.9	7.0 to 13.5

^a^MTB, *Mycobacterium tuberculosis* complex; CI, confidence interval; INH, isoniazid; RMP, rifampicin; EMB, ethambutol; SM, streptomycin.

Drug-resistant TB in countries with good national control programs, such as in Western Europe, is not commonly a major health problem, although increasing immigration prompts public health authorities to maintain vigilant surveillance systems. The results of our study indicate that throughout Italy, prevalence of resistance to firstline drugs and multidrug resistance among isolates from new cases was consistently low over the 4-year survey period. Prevalence of multidrug resistance among isolates from previously treated patients was high, although a downward trend could be demonstrated during the last 2 years. Since almost 2 out of 10 isolates resistant to rifampicin were multidrug resistant, using rapid molecular methods to identify rifampicin resistance in questionable cases appears cost-effective to facilitate early detection and control of multidrug-resistant TB (*10*). Resistance to isoniazid is associated with immigration from countries where isoniazid was used extensively in the past. This information is a useful tool for clinicians, as isoniazid resistance may be suspected early in the disease and properly treated. Finally, the finding of substantial multidrug resistance among isolates from previously treated patients, combined with the evidence that immigrants from areas where isoniazid resistance is endemic contribute substantially to the number of new TB cases in Italy every year, strongly suggests that public health action is needed to improve treatment outcomes.
